# Peripheral Nervous System Function and Organophosphate Pesticide Use among Licensed Pesticide Applicators in the Agricultural Health Study

**DOI:** 10.1289/ehp.1103944

**Published:** 2012-01-19

**Authors:** Sarah E. Starks, Jane A. Hoppin, Freya Kamel, Charles F. Lynch, Michael P. Jones, Michael C. Alavanja, Dale P. Sandler, Fred Gerr

**Affiliations:** 1 Department of Occupational and Environmental Health, University of Iowa, Iowa City, Iowa, USA; 2 Epidemiology Branch, National Institute of Environmental Health Sciences, National Institutes of Health, Department of Health and Human Services, Research Triangle Park, North Carolina, USA; 3 Department of Epidemiology, and; 4 Department of Biostatistics, University of Iowa, Iowa City, Iowa, USA; 5 National Cancer Institute, National Institutes of Health, Department of Health and Human Services, Rockville, Maryland, USA

**Keywords:** agricultural workers, neurological testing, occupational exposure, organophosphates, pesticide exposure

## Abstract

Background: Evidence is limited that long-term human exposure to organophosphate (OP) pesticides, without poisoning, is associated with adverse peripheral nervous system (PNS) function.

Objective: We investigated associations between OP pesticide use and PNS function by administering PNS tests to 701 male pesticide applicators in the Agricultural Health Study (AHS).

Methods: Participants completed a neurological physical examination (NPx) and electrophysiological tests as well as tests of hand strength, sway speed, and vibrotactile threshold. Self-reported information on lifetime use of 16 OP pesticides was obtained from AHS interviews and a study questionnaire. Associations between pesticide use and measures of PNS function were estimated with linear and logistic regression controlling for age and outcome-specific covariates.

Results: Significantly increased odds ratios (ORs) were observed for associations between ever use of 10 of the 16 OP pesticides and one or more of six NPx outcomes. Most notably, abnormal toe proprioception was significantly associated with ever use of 6 OP pesticides, with ORs ranging from 2.03 to 3.06; monotonic increases in strength of association with increasing use was observed for 3 of the 6 pesticides. Mostly null associations were observed between OP pesticide use and electrophysiological tests, hand strength, sway speed, and vibrotactile threshold.

Conclusions: This study provides some evidence that long-term exposure to OP pesticides is associated with signs of impaired PNS function among pesticide applicators.

Acute, high-level exposure to some organophosphate (OP) pesticides is known to result in delayed peripheral neuropathy (Lotti and Moretto 2005); however, the peripheral nervous system (PNS) effects of long-term exposure at levels insufficient to cause clinical toxicity are more controversial. Most research has focused on the central nervous system effects of long-term OP pesticide exposure, with most studies of nonpoisoned individuals reporting increases in neurological symptoms or impaired neurobehavioral function after OP exposure (Kamel and Hoppin 2004). Few studies have examined associations between OP pesticide application and PNS function (Cole et al. 1998; London et al. 1998; Pilkington et al. 2001; Steenland et al. 2000; Stokes et al. 1995), and only one examined associations among participants without prior pesticide poisoning (Steenland et al. 2000). The results of these studies vary, possibly because of differences in exposure characterization, comparison populations and measures of PNS function in addition to small sample sizes (Kamel and Hoppin 2004; Lotti 2002).

The purpose of this epidemiological investigation was to examine cumulative lifetime use of specific OP pesticides and measures of PNS function in a large sample of pesticide applicators with well-characterized lifetime pesticide exposure. The primary hypothesis was that long-term OP pesticide use is associated with adverse peripheral neurological outcomes.

## Methods

*Study population.* We conducted neurological testing on 701 participants in the Agricultural Health Study (AHS), a large prospective study of licensed pesticide applicators from Iowa and North Carolina (Alavanja et al. 1996). Male AHS participants were invited to participate in the present study based on their lifetime use of OPs, completion of earlier parts of the study, and proximity to the testing sites.

The AHS enrolled private pesticide applicators between 1993 and 1997 when they completed a self-administered “enrollment” questionnaire at the time of pesticide licensing and recertification; 44% of private pesticide applicators also completed a take-home questionnaire. These two questionnaires comprised phase 1 data collection. After enrollment, two 5-year follow-up phone interviews were administered (phase 2 and 3 data collection). The present study sample is limited to those who completed all AHS questionnaires. The questionnaires are available online (AHS 2011).

To enrich the sample with applicators with higher lifetime use of OP pesticides, we oversampled the high end of the OP lifetime use distribution based on the lifetime days of use of 10 OPs assessed in detail in phase 1. Among eligible participants, a stratified sample was selected based on equal random sampling from the upper and lower portions of the OP lifetime days distribution. In Iowa, a cut point of approximately 75% was used to separate individuals; in North Carolina the cut point was lower (66%) because the North Carolina cohort is more geographically dispersed and has fewer members. Although the cut point was shifted for selection, all analyses were based on lifetime use of pesticides. Thus, although the sampling frame allowed for a sample enriched for OP use, it was not used as an analytical variable.

For the present study, AHS participants with amyotrophic lateral sclerosis, diabetes, multiple sclerosis, Parkinson’s disease, retinal or macular degeneration, hypothyroidism, and stroke were excluded. In addition, participants who, during phase 3 AHS interviews, reported drinking ≥ 42 alcoholic beverages/week or reported being diagnosed with pesticide poisoning were excluded. After exclusions, a total of 1,807 AHS participants were initially eligible to participate. The overall participation rate was 39%.

In Iowa, testing was conducted in Iowa City and Dubuque between November 2006 and March 2007. In North Carolina, testing was conducted in Greenville and Wilmington between January and March 2008. Institutional review boards approved the study protocol, and all participants provided written informed consent.

*Exposure assessment.* For each participant, pesticide use information from the three AHS phases and a questionnaire administered on the day of neurological testing was used to create ever use and lifetime days of use variables for the 16 OP pesticides used by at least 50 neurological study participants. Ever use was based on any report of use at any interview, and lifetime days of use was based on the sum of lifetime days of use calculated for each interview period.

Pesticide use information was assessed in slightly different ways in each AHS phase. In phase 1 questionnaires, participants were queried in detail about 50 pesticides and asked to provide information on ever use, frequency of use, and years of use. Additionally, on the phase 1 take-home questionnaire, participants were asked to complete a checklist indicating ever use of specific chemicals but were not asked about frequency or duration of use. In phases 2 and 3, participants provided open-ended responses about their pesticide use since the last interview, and their responses were used to create lifetime use information for those time periods. The questionnaire administered on the day of neurological testing collected pesticide use information for the past 12 months. Of the 16 pesticides included in the present study, 9 were reported in detail on the AHS phase 1 questionnaires (chlorpyrifos, coumaphos, diazinon, dichlorvos, fonofos, malathion, parathion, phorate, and terbufos); 6 were initially queried on the phase 1 take-home questionnaire checklist (acephate, dimethoate, disulfoton, ethoprop, phosmet, and tetrachlorvinphos); and a new chemical introduced in 1995 (tebupirimfos) was reported initially on the phase 2 questionnaire.

For phase 1 lifetime days of use, we multiplied the number of days used per year by the numbers of years used to create the summary measure. Because this information was not available for the chemicals listed on the take-home checklist, we assumed that the days per year applied was equal to the median number of days of insecticide use per year for that individual and that the number of years used was equal to the median number of years that individual had applied insecticides using the categories in the phase 1 questionnaire. These values were used to create an estimate of lifetime days of use in phase 1 for the chemicals listed in the take-home checklist. For phases 2 and 3 as well as the neurological study questionnaire, we multiplied the number of days used per year by the number of years since the last interview to create the lifetime days for that period. We then summed the lifetime days for phase 1–3 questionnaires and the neurological study questionnaire to create cumulative lifetime days of use for each pesticide. All pesticide use occurred prior to neurological testing. A summary measure of OP pesticide use (cumulative lifetime days of all OP pesticides) was also created.

We created similar variables for the four carbamate pesticides (aldicarb, benomyl, carbaryl, and carbofuran). In addition, an overall measure of cumulative lifetime pesticide use (all chemical classes) was estimated from all AHS interviews for the 50 pesticides assessed on the AHS enrollment questionnaire. Finally, information on high pesticide exposure events (HPEEs) was obtained from three AHS interviews.

In summary, for every participant, pesticide exposures were characterized as *a*) ever use of 16 OP and 4 carbamate pesticides, *b*) cumulative lifetime days of use for each of these pesticides, *c*) cumulative lifetime days of any OP pesticide use, *d*) cumulative lifetime days of any pesticide use, and *e*) ever having an HPEE.

*Neurological outcomes.* The purpose of the neurological evaluation was to assess neurological function rather than to diagnose clinically apparent peripheral neuropathy.

*Neurological physical examination.* Standard neurological physical examinations (NPxs) (Campbell 2005) were performed by a physician (F.G.) blinded to pesticide exposure status. Assessments of vibration perception (128 Hz) and proprioception were performed on the great toes, bilaterally. Achilles deep tendon reflexes were examined bilaterally, and Romberg test performance, tandem gait, and postural tremor were assessed.

Clinical examination results were recorded as normal, equivocal, or abnormal. For all tests performed bilaterally (ankle reflex, toe proprioception, and toe vibration), examination results were classified as “abnormal” if ratings were abnormal or equivocal bilaterally, abnormal unilaterally and equivocal on the contralateral side, or abnormal unilaterally and missing (because of injury/amputation) on the contralateral side. For tests without laterality (postural tremor, Romberg test, and tandem gait), we combined abnormal and equivocal results to create a dichotomized variable (normal vs. not normal) for each outcome.

*Electrophysiological measures.* Standard noninvasive electrophysiological measures of the dominant peroneal motor nerve were performed with a factory-calibrated TECA Sapphire II electromyograph (TECA Corp., Pleasantville, NY) by one examiner (F.G.) as described by Kimura (2001). Foot temperature was maintained above 32°C during testing. Distal motor amplitude (millivolts), distal and proximal motor latency (milliseconds), and short F-wave latency (milliseconds) were obtained and nerve conduction velocity (meters per second) was calculated.

*Hand strength.* Gross grip strength and key and palmar pinch strength were obtained bilaterally using digital grip and pinch dynamometers (JTech Medical, Salt Lake City, UT) (Mathiowetz et al. 1984). A mean *z*-score for all six hand strength tests (three tests performed bilaterally) combined was calculated to create one summary measure for hand strength.

*Sway speed.* Standing sway speed was measured with a CATSYS 2000 Force Plate (Danish Product Development, Snekkersten, Denmark) (Despres et al. 2000). Four 80-sec trials were administered, two with eyes open and two with eyes closed. Average sway speed (millimeters per second) is reported separately for eyes open and eyes closed.

*Vibrotactile threshold.* The Vibratron II (Sensortek, Inc., Clifton, NJ) was used to measure bilateral great toe 120-Hz vibrotactile threshold with a standard protocol (Gerr et al. 1990). A single bilateral mean vibrotactile threshold is reported in log micrometers.

*Assessment of potential confounders.* Age, height, state (Iowa or North Carolina), smoking status (never, current, or past), education (≤ high school or > high school), alcohol use (0, 1–7, or > 7 drinks/week), ear infection within the past 12 months, prior inner ear surgery, exposure to other potentially neurotoxic substances such as solvents, soldering, and welding fumes (ever used ≥ 8 hr/week), and body mass index (BMI; kilograms per meter squared) were assessed for potential confounding of associations between pesticide use and PNS outcomes.

*Study participant exclusions.* Participants were excluded from all PNS analyses because of past polio (*n* = 6), cancer chemotherapy (*n* = 4), alcohol consumption on the day of testing (*n* = 3), physician diagnosis of alcoholism (*n* = 3), reporting drinking ≥ 42 alcoholic beverages/week during the past year (*n* = 3), diabetes (*n* = 1), dialysis (*n* = 1), severe dementia (*n* = 1), and being struck by lightning (*n* = 1).

Results of all tests except the electrophysiological tests were excluded for 5 participants with a history of brain tumor. For tests of postural stability, we excluded 2 participants who reported current use of the drug meclizine and 2 with Ménière’s disease. Electrophysiological results of 2 participants were excluded after linear regression diagnostics showed studentized residuals with absolute values > 6.0. In addition, a small number of participants were unable to perform certain tests because of recent surgery, amputation, or injury.

*Statistical methods.* Logistic regression was used to estimate odds ratios (ORs) of association between pesticide use and dichotomized NPx results. A base model with no pesticide exposure variables was developed using backward elimination. Adjusted models were run separately for each individual pesticide parameterized as ever use versus never use. Exposure response was examined by creating a three-level variable for individual pesticides with the distribution of lifetime days of use split at the median among the pesticide users to create two exposure categories (≤ median, and > median), with never use as the referent category. The distributions of the pesticide summary variables lifetime days of all OP pesticides and lifetime days of all pesticides were split in quartiles with the lowest category as the referent group. Chi-square tests for trend were used for all exposure–response models with exposure levels assigned 0, 1, and 2 for nonexposed, ≤ the median, and > the median. Analyses were restricted to pesticides with at least five exposed cases per category.

Linear regression was used to examine associations between pesticide use and the continuous outcomes. A base model for each neurological outcome was created with an outcome-specific set of covariates using backward elimination. The final multivariate base model for each outcome included only those covariates with *p*-values < 0.20. Each pesticide was examined both as a dichotomized variable (ever/never use) and as a continuous variable. The cumulative lifetime days of pesticide use variables were log_10_ transformed to normalize the distribution of residuals. Adjusted associations between neurological outcomes and pesticide exposures were estimated with linear regression models in which the neurological outcome was regressed on the pesticide exposure variable while controlling for base model covariates. For greater ease of interpretation, parameter estimates for peroneal nerve distal motor latency and short F-wave latency, sway speed, and vibrotactile threshold were multiplied by –1 so that lower scores indicated poorer performance for all continuous outcomes.

We examined potential confounding of the association between neurological outcomes and each pesticide by other pesticides in both linear and logistic regression models. Specifically, pesticide pairs with *r* ≥ 0.30 were added simultaneously to final base models, and the pesticide variable parameter estimates were compared with models with one pesticide.

To evaluate whether associations between pesticide use and neurological outcomes were influenced by previous pesticide poisoning, the 8 participants who reported ever being physician diagnosed with pesticide poisoning at the time of AHS enrollment were excluded from the analyses, and the results were compared with models that included them. The results did not change.

All analyses were performed using SAS software (version 9.2; SAS Institute Inc., Cary, NC).

## Results

A total of 701 individuals participated in neurological testing; 23 were excluded, resulting in 678 included in these analyses. The mean ± SD age of the participants was 61 ± 11.6 years (Table 1). Approximately half of the participants reported completing at least a high school education. Nearly all participants reported ever using any OP [98%; see Supplemental Material, Table 1 (http://dx.doi.org/10.1289/ehp.1103944)]. Proportions of applicators reporting use of specific OPs ranged from 77% for malathion to < 10% for dimethoate, tebupirimfos, and tetrachlorvinphos. NPx signs and electrophysiological and quantitative functional PNS test results were consistent with clinical expectation for the age of the sample. Descriptive statistics for these outcomes are presented in Supplemental Material, Table 2.

**Table 1:** Demographic characteristics, personal health information, and occupational exposure among 678 male licensed pesticide applicators in the AHS.

Characteristic	Mean ± SD or *n* (%)
Age (years)	61.2 ± 11.6
Height (cm)	179.1 ± 6.4
BMI (kg/m^2^)	28.7 ± 4.0
Foot temperature (°C)	31.9 ± 0.8
Testing location	
Iowa	342 (50.4)
North Carolina	336 (49.6)
Education	
≤ High school	344 (50.7)
> High school	334 (49.3)
Smoking status	
Never smoked	390 (57.5)
Current smoker	43 (6.3)
Past smoker	245 (36.1)
Alcohol consumption*a*	
0 drinks	389 (57.4)
1–7 drinks	225 (33.2)
> 7 drinks	64 (9.4)
Pesticide poisoning	8 (1.2)
Solvent exposure*b*	279 (41.2)
Soldering exposure*b*	34 (5.0)
Welding exposure*b*	136 (20.1)
Inner ear surgery	14 (2.1)
Ear infection in the previous 12 months	17 (2.5)
**a**The average number of drinks/week during the past 12 months. **b**Ever worked ≥ 8 hr/week at a past or present job or at home that resulted in exposure.	

**Table 2:** Adjusted ORs (95% CIs) between NPx tests and ever use of individual pesticides among 678 male pesticide applicators in the AHS.

Exposure	Ankle reflex (abnormal = 109, normal = 554)	Postural tremor (abnormal = 117, normal = 547)	Romberg (abnormal = 59, normal = 586)	Tandem gait (abnormal = 180, normal = 586)	Toe proprioception (abnormal = 62, normal = 603)	Toe vibration (abnormal = 120, normal = 554)
OPs												
Acephate		0.67 (0.39, 1.16)		1.39 (0.89, 2.19)		0.84 (0.34, 2.08)		0.60 (0.37, 0.97)		—		0.54 (0.25, 1.18)
Chlorpyrifos		0.81 (0.52, 1.26)		1.03 (0.68, 1.56)		1.43 (0.78, 2.62)		1.21 (0.81, 1.82)		2.35 (1.28, 4.31)		1.45 (0.91, 2.30)
Coumaphos		1.40 (0.75, 2.63)		0.93 (0.50, 1.74)		1.12 (0.50, 2.54)		1.05 (0.60, 1.89)		2.03 (1.06, 3.90)		1.27 (0.70, 2.31)
Diazinon		1.24 (0.80, 1.93)		0.63 (0.41, 0.96)		0.69 (0.38, 1.26)		1.07 (0.72, 1.58)		0.67 (0.39, 1.16)		1.02 (0.65, 1.62)
Dichlorvos		1.35 (0.78, 2.34)		0.81 (0.46, 1.40)		1.06 (0.50, 2.23)		2.29 (1.41, 3.71)		2.73 (1.53, 4.86)		1.94 (1.32, 3.31)
Dimethoate		1.20 (0.58, 2.48)		1.90 (1.01, 3.54)		0.94 (0.34, 2.59)		1.26 (0.66, 2.43)		1.86 (0.86, 4.00)		1.66 (0.83, 3.31)
Disulfoton		0.95 (0.53, 1.70)		1.95 (1.19, 3.18)		0.84 (0.33, 2.15)		0.80 (0.47, 1.35)		0.39 (0.15, 1.01)		0.67 (0.30, 1.50)
Ethoprop		1.03 (0.58, 1.80)		2.16 (1.35, 3.47)		1.52 (0.65, 3.58)		0.98 (0.59, 1.64)		0.36 (0.14, 0.93)		1.08 (0.52, 2.23)
Fonofos		1.47 (0.92, 2.34)		1.07 (0.69, 1.66)		0.90 (0.45, 1.81)		1.18 (0.77, 1.80)		3.06 (1.79, 5.25)		1.62 (0.98, 2.68)
Malathion		1.37 (0.78, 2.34)		0.88 (0.54, 1.42)		0.73 (0.37, 1.43)		0.82 (0.51, 1.31)		1.05 (0.55, 2.00)		1.20 (0.69, 2.09)
Parathion		0.95 (0.57, 1.60)		0.99 (0.61, 1.61)		0.72 (0.34, 1.50)		0.73 (0.46, 1.16)		0.34 (0.14, 0.81)		1.30 (0.75, 2.25)
Phorate		0.68 (0.42, 1.11)		0.81 (0.52, 1.26)		0.86 (0.45, 1.63)		1.05 (0.70, 1.59)		1.67 (0.97, 2.84)		0.99 (0.61, 1.60)
Phosmet		2.87 (1.52, 5.44)		0.64 (0.31, 1.29)		1.18 (0.46, 3.01)		1.71 (0.93, 3.12)		2.82 (1.47, 5.42)		0.73 (0.38, 1.41)
Tebupirimfos		2.01 (1.00, 4.05)		2.17 (1.18, 4.00)		—		1.00 (0.51, 1.95)		1.84 (0.85, 4.01)		0.86 (0.42, 1.76)
Terbufos		0.95 (0.61, 1.48)		0.89 (0.59, 1.34)		0.79 (0.42, 1.47)		1.08 (0.73, 1.59)		1.25 (0.73, 2.13)		1.02 (0.64, 1.62)
Tetrachlorvinphos		1.13 (0.53, 2.34)		1.36 (0.70, 2.62)		1.68 (0.67, 4.24)		1.01 (0.50, 2.02)		2.35 (1.11, 4.98)		2.15 (1.11, 4.17)
Carbamates												
Aldicarb		0.43 (0.22, 0.86)		1.37 (0.84, 2.26)		0.46 (0.16, 1.31)		1.00 (0.61, 1.65)		0.43 (0.18, 1.02)		1.09 (0.53, 2.26)
Benomyl		0.65 (0.35, 1.22)		1.26 (0.75, 2.11)		0.87 (0.35, 2.18)		0.52 (0.30, 0.90)		0.40 (0.15, 1.02)		1.28 (0.64, 2.59)
Carbaryl		0.67 (0.42, 1.06)		1.39 (0.89, 2.16)		0.74 (0.38, 1.45)		0.77 (0.51, 1.17)		0.45 (0.26, 0.77)		1.25 (0.75, 2.07)
Carbofuran		0.77 (0.50, 1.21)		0.95 (0.63, 1.42)		0.48 (0.26, 0.90)		0.95 (0.64, 1.41)		1.45 (0.85, 2.46)		1.23 (0.79, 1.93)
HPEEs		0.96 (0.56, 1.67)		1.29 (0.80, 2.08)		1.57 (0.78, 3.15)		1.03 (0.63, 1.68)		0.99 (0.51, 1.90)		0.68 (0.38, 1.21)
Ankle reflex models were adjusted for age and BMI; postural tremor models were adjusted for age; Romberg models were adjusted for age, height, and state; tandem gait models were adjusted for age and height; toe proprioception models were adjusted for age and height; toe vibration models were adjusted for age, height, and state. Results from models with fewer than five exposed cases are not presented.												

*Base model covariates.* Age was included in all neurological outcome models, and height was included in all models except for ankle reflex and postural tremor. The total variance (*R*^2^) accounted for by linear regression models ranged from 0.16 for distal motor amplitude to 0.40 for hand strength. Base model covariates for the linear and logistic regression models are presented in Supplemental Material, Table 3 (http://dx.doi.org/10.1289/ehp.1103944).

**Table 3:** Adjusted regression coefficients [β (95% CI)] between electrophysiological tests and pesticide use (ever use and log_10_ lifetime days of use) among 678 male pesticide applicators in the AHS.

Exposure	Distal motor amplitude (mV; *n* = 664)	Distal motor latency (msec; *n* = 655)*a*	Nerve conduction velocity (m/sec; *n* = 652)	Short F-wave latency (msec; *n* = 544)*a*
OPs				
Acephate				
Ever use	0.01 (–0.53, 0.54)	0.03 (–0.14, 0.20)	0.42 (–0.42, 1.27)	1.18 (0.20, 2.16)
Lifetime days	0.05 (–0.24, 0.34)	0.02 (–0.07, 0.11)	0.22 (–0.24, 0.68)	0.73 (0.21, 1.25)
Chlorpyrifos				
Ever use	0.05 (–0.33, 0.43)	–0.07 (–0.20, 0.05)	0.12 (–0.48, 0.73)	0.09 (–0.60, 0.79)
Lifetime days	–0.02 (–0.24, 0.19)	–0.01 (–0.08, 0.05)	0.18 (–0.16, 0.53)	0.11 (–0.28, 0.50)
Coumaphos				
Ever use	–0.09 (–0.64, 0.46)	–0.04 (–0.22, 0.13)	0.35 (–0.53, 1.24)	0.36 (–0.64, 1.37)
Lifetime days	–0.05 (–0.43, 0.34)	–0.01 (–0.14, 0.11)	0.25 (–0.37, 0.87)	0.00 (–0.68, 0.68)
Diazinon				
Ever use	0.04 (–0.34, 0.42)	–0.12 (–0.24, 0.00)	0.07 (–0.54, 0.68)	0.09 (–0.61, 0.80)
Lifetime days	0.08 (–0.16, 0.33)	–0.07 (–0.15, 0.00)	0.07 (–0.33, 0.46)	0.16 (–0.32, 0.58)
Dichlorvos				
Ever use	–0.31 (–0.82, 0.20)	–0.15 (–0.32, –0.01)	–0.14 (–0.96, 0.67)	–0.66 (–1.57, 0.26)
Lifetime days	–0.03 (–0.27, 0.21)	–0.05 (–0.13, 0.02)	–0.11 (–0.49, 0.27)	–0.27 (–0.70, 0.15)
Dimethoate				
Ever use	0.23 (–0.40, 0.86)	–0.02 (–0.21, 0.18)	–0.07 (–1.07, 0.92)	–0.04 (–1.20, 1.12)
Lifetime days	0.10 (–0.33, 0.52)	–0.01 (–0.15, 0.12)	–0.01 (–0.68, 0.66)	–0.08 (–0.86, 0.70)
Disulfoton				
Ever use	0.03 (–0.53, 0.59)	0.07 (–0.11, 0.25)	–0.40 (–1.30, 0.49)	0.57 (–0.47, 1.61)
Lifetime days	0.01 (–0.35, 0.37)	0.03 (–0.09, 0.14)	–0.22 (–0.80, 0.36)	0.46 (–0.21, 1.14)
Ethoprop				
Ever use	–0.41 (–0.95, 0.12)	–0.08 (–0.25, 0.09)	–0.60 (–1.45, 0.24)	–0.01 (–1.01, 0.99)
Lifetime days	–0.28 (–0.62, 0.07)	–0.06 (–0.17, 0.05)	–0.36 (–0.91, 0.19)	–0.08 (–0.72, 0.57)
Fonofos				
Ever use	0.05 (–0.40, 0.50)	–0.09 (–0.24, 0.05)	0.09 (–0.63, 0.81)	–0.09 (–0.91, 0.73)
Lifetime days	0.06 (–0.21, 0.33)	–0.04 (–0.13, 0.04)	–0.04 (–0.48, 0.39)	–0.05 (–0.54, 0.44)
Malathion				
Ever use	0.28 (–0.16, 0.72)	–0.02 (–0.16, 0.12)	–0.09 (–0.79, 0.61)	–0.70 (–1.51, 0.11)
Lifetime days	–0.03 (–0.25, 0.19)	–0.05 (–0.12, 0.02)	0.01 (–0.34, 0.36)	–0.27 (–0.67, 0.14)
Parathion				
Ever use	0.22 (–0.25, 0.68)	–0.07 (–0.22, –0.08)	–0.01 (–0.75, 0.73)	0.31 (–0.53, 1.15)
Lifetime days	0.25 (–0.04, 0.54)	0.01 (–0.08, 0.10)	0.03 (–0.43, 0.49)	0.17 (–0.34, 0.68)
Phorate				
Ever use	0.47 (0.06, 0.88)	0.01 (–0.13, 0.14)	0.64 (–0.01, 1.29)	0.91 (0.16, 1.66)
Lifetime days	0.28 (0.04, 0.52)	0.01 (–0.06, 0.09)	0.51 (0.12, 0.89)	0.73 (0.29, 1.17)
Phosmet				
Ever use	–0.07 (–0.63, 0.49)	–0.05 (–0.23, 0.13)	–0.21 (–1.11, 0.69)	–0.27 (–1.30, 0.75)
Lifetime days	–0.09 (–0.43, 0.25)	–0.01 (–0.13, 0.10)	–0.10 (–0.65, 0.46)	–0.04 (–0.68, 0.59)
Tebupirimfos				
Ever use	0.71 (0.05, 1.37)	0.06 (–0.15, 0.27)	0.78 (–0.28, 1.84)	–0.05 (–1.24, 1.13)
Lifetime days	0.43 (0.02, 0.84)	0.04 (–0.09, 0.17)	0.51 (–0.14, 1.17)	0.08 (–0.65, 0.80)
Terbufos				
Ever use	0.20 (–0.18, 0.59)	0.05 (–0.07, 0.17)	0.21 (–0.41, 0.82)	0.16 (–0.54, 0.86)
Lifetime days	0.14 (–0.06, 0.34)	0.04 (–0.03, 0.10)	0.18 (–0.15, 0.51)	0.30 (–0.07, 0.67)
Tetrachlorvinphos				
Ever use	–0.40 (–1.03, 0.24)	–0.05 (–0.24, 0.15)	–0.04 (–1.06, 0.99)	–0.36 (–1.50, 0.77)
Lifetime days	–0.23 (–0.62, 0.16)	–0.02 (–0.14, 0.10)	0.15 (–0.47, 0.77)	–0.13 (–0.81, 0.56)
Carbamates				
Aldicarb				
Ever use	0.30 (–0.22, 0.83)	–0.06 (–0.22, 0.11)	–0.61 (–0.23, 1.44)	1.73 (0.74, 2.71)
Lifetime days	0.18 (–0.12, 0.49)	–0.06 (–0.16, 0.03)	0.27 (–0.21, 0.75)	0.87 (0.31, 1.44)
Benomyl				
Ever use	0.28 (–0.26, 0.82)	–0.06 (–0.23, 0.11)	0.62 (–0.23, 1.48)	0.60 (–0.40, 1.60)
Lifetime days	0.11 (–0.24, 0.47)	–0.07 (–0.18, 0.04)	0.44 (–0.12, 1.00)	0.39 (–0.27, 1.05)
Carbaryl				
Ever use	–0.10 (–0.54, 0.33)	–0.13 (–0.26, 0.01)	–0.30 (–0.99, 0.40)	–0.62 (–1.40, 0.16)
Lifetime days	0.08 (–0.16, 0.33)	–0.10 (–0.18, –0.02)	–0.01 (–0.41, 0.38)	–0.31 (–0.76, 0.13)
Carbofuran				
Ever use	–0.03 (–0.41, 0.35)	–0.07 (–0.20, 0.04)	–0.18 (–0.78, 0.43)	–0.30 (–0.99, 0.40)
Lifetime days	0.00 (–0.24, 0.23)	–0.04 (–0.11, 0.04)	0.03 (–0.35, 0.41)	–0.08 (–0.52, 0.36)
Summary variables				
Lifetime days to all OPs	0.22 (–0.06, 0.49)	–0.02 (–0.10, 0.07)	0.31 (–0.12, 0.74)	0.53 (0.02, 1.02)
Lifetime days to all pesticides	0.19 (–0.19, 0.58)	0.10 (–0.22, 0.02)	0.53 (–0.08, 1.14)	0.75 (0.01, 1.50)
HPEEs (ever)	0.54 (0.10, 0.98)	–0.01 (–0.15, 0.13)	0.02 (–0.68, 0.72)	0.16 (–0.63, 0.96)
Electrophysiological tests were adjusted for age, height, foot temperature, and state. **a**Scores have been multiplied by –1 so that lower scores indicate poorer test results.				

*NPx results.* Overall, ever use of 10 of the 16 OP pesticides was associated with one or more of the six NPx outcomes (Table 2). Six OPs were associated with toe proprioception abnormality, and 1 OP was inversely associated with toe proprioception abnormality [i.e., upper bound of the 95% confidence interval (CI) ≤ 1.0]. Four OPs were associated with postural tremor abnormality, and 1 OP was inversely associated with postural tremor abnormality. The only overlap between these two outcomes was for ethoprop, which was associated with postural tremor abnormality and inversely associated with toe proprioception abnormality. The three-level exposure response models were generally consistent with the ever-use models [see Supplemental Material, Table 4 (http://dx.doi.org/10.1289/ehp.1103944)]. All 4 carbamate pesticides were inversely associated with NPx abnormalities. No significant associations were observed between NPx results and lifetime days of all OP pesticides or lifetime days of all pesticides (data not shown). Results for each NPx outcome are presented below.

*Ankle reflex.* Ever use of phosmet and of tebupirimfos were significantly associated with ankle reflex abnormality, whereas ever use of aldicarb was inversely associated with ankle reflex abnormality. In dose–response models, a significant monotonic increase in ORs was observed for fonofos and phosmet. There was insufficient data to evaluate tebupirimfos in dose–response models. There was no evidence of a monotonic dose response for aldicarb.

*Postural tremor.* Elevated ORs were observed for ever use of dimethoate, disulfoton, ethoprop, and tebupirimfos and postural tremor abnormality. Tests for trend were significant for dimethoate, disulfoton, ethoprop, and tebupirimfos, whereas the increase in ORs for disulfoton was not monotonic. Although ever use of diazinon was associated with a reduced OR, there was no evidence of a dose response.

*Romberg test.* We observed an inverse association for ever use of carbofuran and Romberg test abnormality with no significant test for trend.

*Tandem gait.* Dichlorvos use was associated with tandem gait abnormality, and whereas the test for trend was significant, the increase across ORs was not monotonic. Although an elevated but nonsignificant OR for ever use of phosmet was observed, a significant dose–response trend was also observed. Ever use of acephate and benomyl were both inversely associated with tandem gait abnormality, but neither showed a dose response.

*Toe proprioception.* Ever use of chlorpyrifos, coumaphos, dichlorvos, fonofos, phosmet, and tetrachlorvinphos were associated with toe proprioception abnormality, whereas ORs among users of carbaryl and ethoprop were below unity. In the dose–response models, chlorpyrifos, dichlorvos, fonofos, phorate, phosmet, and carbaryl all had significant tests for trend, and all but dichlorvos showed a monotonic increase in ORs.

*Toe vibration.* Significant adverse associations were observed with ever use of dichlorvos and tetrachlorvinphos and toe vibration. Dichlorvos also had a significant dose response for trend. There were too few counts to evaluate the dose response for tetrachlorvinphos.

*Electrophysiological measures*. Adjusted regression coefficients for peroneal motor nerve electrophysiological measures and pesticide use are presented in Table 3. Overall, we observed more associations consistent with reduced risk (i.e., positive parameter estimates); however, few were statistically significant. Reduced risk estimates were observed between distal motor amplitude and phorate, tebupirimfos, and HPEEs. The dose–response models for phorate and tebupirimfos were consistent with a monotonic risk reduction for distal motor amplitude. Phorate was associated with faster nerve conduction velocity. Acephate, phorate, aldicarb, lifetime days of all OPs, and lifetime days of all pesticides showed shorter F-wave latency (i.e., lower risk) with increasing pesticide use. Only distal motor latency showed increased risk with pesticide use. Greater distal motor latency (i.e., greater risk) was observed among users of diazinon and carbaryl, with risk increasing with increasing use.

*Quantitative functional measures.* We found no significant increases in risk between quantitative functional PNS tests and pesticide use [see Supplemental Material, Table 5 (http://dx.doi.org/10.1289/ehp.1103944)]. However, ever use and lifetime days of phosmet use and HPEE were associated with greater hand strength. Both measures of parathion, tebupirimfos, and aldicarb use were associated with lower sway speed (i.e., better sway function) with eyes open. However, the association with lower sway speed with eyes closed was limited to aldicarb. Aldicarb was also associated with lower (better) vibrotactile threshold.

*Confounding by correlated pesticide exposures.* Dichlorvos and coumaphos use were correlated in our sample. In models of toe proprioception that included both pesticides, the OR for coumaphos and toe proprioception was attenuated from 2.03 to 1.44 (95% CI: 0.71, 2.92), whereas the OR for dichlorvos decreased by < 10%, from 2.73 to 2.45 (95% CI: 1.32, 4.54). We observed no other evidence of confounding by multiple pesticides.

## Discussion

We observed significant associations between use of several OP pesticides and NPx abnormalities among pesticide applicators. No single pesticide was uniformly associated with all neurological measures, and no one measure was uniformly associated with all pesticides. Toe proprioception was the most responsive NPx outcome and was associated with 6 of 16 OP pesticides. Significant exposure–response relationships were observed for several pesticides, suggesting that associations between these pesticides and toe proprioception abnormality were not spurious. For most other outcomes, we observed mostly null associations and a few significant inverse associations between OP pesticide use and other PNS outcomes. Despite the inconsistency in findings across the outcome measures, our results provide some evidence that long-term exposure to specific OP pesticides may adversely affect the PNS.

Published studies show considerable variability in association between OP pesticide exposure and PNS outcomes. Studies of PNS function among pesticide workers vary with respect to the populations studied (manufacturing workers to pesticide applicators), outcomes assessed (e.g., neurological signs and symptoms, electrophysiological tests), exposure characterization, inclusion of pesticide-poisoned individuals, and age of participants. In a study of 123 OP pesticide applicators (mean age, 36 years) and 123 nonapplicators (mean age, 37 years), significantly elevated ORs were observed for motor coordination signs, deep tendon reflexes, and reduced muscle strength among the most heavily exposed applicators (Cole et al. 1998). Contrary to our results, Cole et al. (1998) observed a borderline association for vibrotactile threshold but not for toe vibration abnormality on NPx. In a study of 164 OP pesticide applicators (mean age = 34 years) and 83 unexposed controls (mean age, 33 years), neither vibrotactile threshold nor tremor was associated with pesticide exposure (London et al. 1998). In a study comparing 191 OP termiticide applicators (mean age, 39 years) with 106 unexposed friends (mean age, 38 years) and 83 unexposed workers (mean age, 43 years), no difference was observed between applicators and either comparison group on NPx outcomes or measures of great toe vibrotactile threshold (Steenland et al. 2000). However, unlike the present study, mean sway path length was significantly longer among the applicators.

PNS symptoms were assessed with a questionnaire and quantitative measures of vibrotactile and thermal thresholds were administered to 612 “sheep-dipping” farmers exposed to OP pesticides (mostly < 55 years of age) and 160 unexposed referents (Pilkington et al. 2001). Neurological symptoms were not associated with total number of dipping days or cumulative OP exposure. Cumulative OP exposure was not associated with cutaneous temperature perception or vibrotactile threshold. Handling of OP concentrate was associated with symptoms and marginally associated with cutaneous cold temperature perception.

Vibrotactile thresholds were measured among 68 male pesticide applicators in New York State (mostly < 50 years of age) and 68 referents matched for age, sex, and county of residence (Stokes et al. 1995). Azinphos-methyl was the most commonly used insecticide. Poorer vibrotactile acuity of the fingers and toes was observed among the applicators.

In contrast to earlier studies of OP exposure and PNS function, we focused on exposure to specific OPs and on lifetime exposure to any OPs. Other studies collected data on specific pesticide exposures but ultimately presented data only for summary variables. For example, although Cole et al. (1998) characterized exposure to OP and carbamate insecticides and dithiocarbamate fungicides among Ecuadorian agricultural workers, their analyses were limited to comparisons of current pesticide applicators with nonexposed referents. Similarly, Stokes et al. (1995) collected questionnaire-based information about lifetime use of specific OPs among pesticide applicators in New York but reported only comparisons of applicators with referents.

In the present study, we focused on specific chemical exposures, rather than comparing pesticide applicators with nonapplicators; this allowed us to further explore whether specific OPs contributed to adverse PNS function. We estimated cumulative lifetime exposure based on self-reported pesticide use information reported at four points in time. We relied on this metric of lifetime exposure as our primary exposure metric. In general, our results were similar for both ever use of a specific OP and continuous days of use. The fact that the continuous results were not different from the ever use estimates may be a result of the assumptions used to create the continuous exposure measure (e.g., potentially overestimating exposure and thus attenuating the exposure–response relationship), or it may be that the assumption of chronic exposure as the most relevant exposure was incorrect and, in fact, intermittent higher level exposures contributed more to increased risk. However, such intermittent exposures are not well quantified using our questionnaire. Or it may be that using a log transform of the exposure metric attenuated the impact of the high-end values, thus making it more difficult to detect associations limited to the high end of the distribution. Although the estimated metrics may be limited because of the assumptions, methodological studies have shown that AHS participants provide accurate and reliable pesticide use and duration of pesticide exposure information (Blair et al. 2002; Hoppin et al. 2002).

Another important difference between our study and those conducted previously is the age of the workers. The average age of participants in our study was 61 years, whereas the average age of participants in earlier studies ranged from 30 to approximately 55 years. It is possible that age or exposure duration potentiates the effects of exposure and accounts for differences between studies. Also, contrary to the present study, none of the studies reviewed reported exclusion of participants with a past episode of pesticide poisoning.

In summary, the existing literature is relatively small and inconsistent in methods and results. Important questions remain regarding the health outcomes most responsive to exposure effects, possible differential effects of specific chemicals, and the actual pathophysiological process responsible for observed impairments.

One interesting finding of the present study was the observation of stronger associations between pesticide use and NPx outcomes than with the analogous quantitative measures. For example, we observed significant adverse associations between abnormal toe vibration on NPx and two OP pesticides (dichlorvos and tetrachlorvinphos), but we did not observe associations between quantitative toe vibrotactile thresholds and these chemicals. Our *a priori* expectation was that quantitative measures would be as sensitive as NPx to PNS effects. Because the examiner was blinded to pesticide exposure status, we do not believe that observer bias accounts for these findings. It is possible that, despite considerable training of quantitative test administrators, nondifferential error was greater for the quantitative tests than for the NPx outcomes. Another possibility is that the NPx better captured relevant peripheral nerve impairment than did the quantitative measures.

Eligibility for the present study required completion of all AHS questionnaires, and individuals who were no longer farming at enrollment were less likely to meet this requirement (Montgomery et al. 2010). The reasons for leaving farming are many, but those with peripheral nerve impairment may have been more likely to leave farming than those without. Thus, it is possible that loss from the sample of those most susceptible to the adverse effects of pesticide exposure attenuated the observed associations. Such selective survival may also lead to the inclusion of healthy older participants with longer pesticide use durations, creating the appearance of an association between pesticide use and improved PNS function. Given the response rate of 39%, our sample may not have been representative of all AHS participants. However, on several important characteristics, including age and total lifetime days of pesticide use, participants were similar to eligible nonparticipants, suggesting comparability between them.

Given the data complexity of 14 outcomes and > 100 exposures, we presented the results of numerous statistical tests. No formal correction for multiple comparisons was made, but efforts were made to reduce the number of outcomes where appropriate. For example, we combined six hand strength measures into a single hand strength metric, and we averaged the vibrotactile thresholds of the dominant and nondominant great toes. However, given that we performed hundreds of statistical tests and observed significant associations in both positive and negative directions, some of our findings may be due to chance.

Previous studies of PNS function among pesticide applicators have included participants with past pesticide poisoning (28%, Cole et al. 1998; 9%, London et al. 1998), a known cause of peripheral neuropathy (Lotti and Moretto 2005). Associations were typically stronger among poisoned than among nonpoisoned participants. Because we did not recruit participants who reported a past diagnosis of pesticide poisoning at the most recent AHS interview, it is unlikely that adverse associations resulted from previous pesticide poisoning. Furthermore, our findings were unchanged by exclusion of the eight participants who reported a past diagnosis of pesticide poisoning at the time of AHS enrollment. It is also unlikely that current pesticide toxicity accounted for our results because evaluations were performed during the winter when pesticides were not applied. In addition to pesticides, pesticide applicators are potentially exposed to a number of other neurotoxicants, such as organic solvents and soldering and welding fumes. We assessed these agents at the time of neurological testing and evaluated whether they confounded the pesticide associations. We observed no evidence of confounding of any PNS measure by these occupational exposures; however, we cannot rule out the possibility of confounding by unmeasured neurotoxicants.

In this study of 678 licensed pesticide applicators with well-characterized lifetime exposure to OP pesticides, our findings provide evidence that long-term exposure to some OP pesticides may be associated with selected indices of poorer PNS function among pesticide applicators with no previous history of diagnosed pesticide poisoning. The most consistent associations were with the clinical NPx outcomes, particularly toe proprioception, which was significantly associated with ever use of chlorpyrifos, coumaphos, dichlorvos, fonofos, phosmet, and tetrachlorvinphos. Furthermore, monotonic increases in ORs were observed for abnormal toe proprioception and chlorpyrifos, fonofos, and phosmet. Especially for this outcome, the number of pesticides with significant adverse associations and the observation of dose–response relationships suggest that they are the result of long-term pesticide exposure rather than chance alone.

## Supplemental Material

(201 KB) PDF
