# Supplementary material for: Peripheral Nervous System Function and Organophosphate Pesticide Use among Licensed Pesticide Applicators in the Agricultural Health Study
Source: Environ Health Perspect. 2012 Jan 19;120(4):515–20. doi: 10.1289/ehp.1103944 (PMC3339452; doi:10.1289/ehp.1103944)
Supplement: (201 KB) PDF [file ehp.1103944.s001.pdf]

SUPPLEMENTAL MATERIAL

PERIPHERAL NERVOUS SYSTEM FUNCTION AND ORGANOPHOSPHATE PESTICIDE USE AMONG LICENSED PESTICIDE APPLICATORS IN THE AGRICULTURAL HEALTH STUDY

Sarah E Starks, Jane A Hoppin, Freya Kamel, Charles F Lynch, Michael P Jones, Michael C Alavanja, Dale P Sandler, Fred Gerr

TABLE OF CONTENTS

Page 2. Supplemental Table 1. Pesticide use among 678 male licensed pesticide applicators.

Page 3. Supplemental Table 2. Descriptive statistics for neurological physical examination tests, electrophysiological tests and quantitative functional PNS tests among 678 male licensed pesticide applicators.

Page 4. Supplemental Table 3. Logistic and linear regression base model covariates for peripheral nervous system (PNS) outcome measures.

Page 5. Supplemental Table 4. Results from dose-response models of neurological physical examination tests among 678 male pesticide applicators.

Page 8. Supplemental Table 5. Adjusted regression coefficients for quantitative functional PNS tests and pesticide use (ever-use and $\log_{10}$ lifetime days of use) among 678 male licensed pesticide applicators in the Agricultural Health Study. .

Supplemental Material, Table 1. Pesticide use among 678 male licensed pesticide applicators

Pesticide exposure	N*	%	Mean	SD	Min.	Median	Max.
Organophosphates							
Acephate	163	24.0	86.2	90.9	2.5	56.0	500.5
Chlorpyrifos	406	59.9	74.3	102.6	2.0	38.8	767.3
Coumaphos	90	13.3	76.1	250.6	1.0	10.3	1,682.5
Diazinon	294	43.4	55.6	94.1	1.0	20.9	846.0
Dichlorvos	123	18.1	454.0	1,083.5	1.0	56.0	8,680.0
Dimethoate	64	9.4	47.1	69.2	2.0	24.5	457.3
Disulfoton	107	15.8	42.9	42.1	2.0	24.5	236.0
Ethoprop	119	17.6	45.5	49.8	2.5	24.5	316.0
Fonofos	195	28.8	63.6	82.3	2.0	38.8	457.3
Malathion	525	77.4	99.8	203.8	2.0	37.0	2,625.0
Parathion	143	21.1	103.3	274.9	1.0	20.0	1,667.5
Phorate	218	32.2	71.7	132.6	2.0	27.0	1,627.5
Phosmet	99	14.6	61.6	83.7	2.5	27.3	600.0
Tebupirimfos	64	9.4	50.7	47.0	4.0	39.5	250.0
Terbufos	344	50.7	100.6	116.1	2.0	56.0	752.3
Tetrachlorvinphos	66	9.7	66.7	100.9	3.0	24.5	581.6
Carbamates							
Aldicarb	127	18.7	88.7	119.0	2.0	29.5	742.3
Benomyl	112	16.5	63.7	123.7	1.0	15.4	767.3
Carbaryl	430	63.4	103.4	153.2	1.0	47.5	1,387.5
Carbofuran	281	41.5	56.3	93.1	1.0	24.5	752.3
Summary variables							
All organophosphates	661	97.5	423.6	671.9	2.0	240.8	8,763.3
All pesticides	677	99.9	1,619.5	1,634.4	10.0	1,045.5	11,676.8
HPEEs (ever)**	158	23.3	--	--	--	--	--

* Number of participants who reported ever-use.

** HPEEs, high pesticide exposure events

Supplemental Material, Table 2. Descriptive statistics for neurological physical examination tests, electrophysiological tests and quantitative functional PNS tests among 678 male licensed pesticide applicators

Outcome	N	Abnormal N	Abnormal %	Mean	SD	Min.	Max.
Neurological physical examination							
Ankle reflex	663	109	16.4	--	--	--	--
Postural tremor	664	117	17.6	--	--	--	--
Romberg	645	59	9.2	--	--	--	--
Tandem gait	641	180	28.1	--	--	--	--
Toe proprioception	665	62	9.3	--	--	--	--
Toe vibration	664	120	18.1	--	--	--	--
Electrophysiological tests*							
Distal motor amplitude (mV)	665	--	--	4.9	2.6	0.0	13.9
Distal motor latency (ms)	656	--	--	5.1	0.9	3.3	9.3
Nerve conduction velocity (m/s)	653	--	--	44.1	4.4	25.0	56.8
Short F-wave latency (ms)	545	--	--	53.0	5.0	42.1	77.9
Quantitative functional PNS tests							
Hand strength (kg)							
Grip, d.	666	--	--	38.2	9.6	9.6	72.9
Grip, nd.	668	--	--	38.2	9.9	8.7	70.9
Key pinch, d.	665	--	--	10.9	2.0	1.3	16.5
Key pinch, nd.	667	--	--	10.8	2.1	2.7	19.7
Palmar pinch, d.	665	--	--	9.9	2.3	0.7	16.7
Palmar pinch, nd.	667	--	--	10.1	2.4	1.1	20.3
Sway speed - eyes open (mm/s)	655	--	--	14.6	5.1	5.0	38.6
Sway speed - eyes closed (mm/s)	656	--	--	22.9	10.2	6.4	70.6
Vibrotactile threshold, d. (log µ)	660	--	--	1.5	0.5	-0.4	2.4
Vibrotactile threshold, nd. (log µ)	661	--	--	1.5	0.5	-1.0	2.4

d, dominant; nd, non-dominant; µ, microns

* Electrophysiological tests were performed on the peroneal motor nerve.

Supplemental Material, Table 3. Logistic and linear regression base model covariates for peripheral nervous system (PNS) outcome measures

Outcome	N	Age (yrs)	BMI (kg/m^2)	Height (cm)	Foot temp. (°C)	State (N. Carolina)	Model R-square*
<u>Logistic regression results (odds ratios)</u>							
Neurological physical examinations							
Ankle reflex	663	1.09†	1.11†	--	--	--	--
Postural tremor	664	1.04†	--	--	--	--	--
Romberg	645	1.12†	--	1.07†	--	0.43†	--
Tandem gait	641	1.11†	--	1.05†	--	--	--
Toe proprioception	665	1.03†	--	1.05†	--	--	--
Toe vibration	664	1.09†	--	1.13†	--	0.22†	--
<u>Linear regression results (parameter estimates)</u>							
Electrophysiological tests							
Distal motor amplitude (mV)	664	-0.08†	--	-0.08†	-0.33†	0.60†	0.16
Distal motor latency (ms)**	655	-0.01†	--	-0.02†	0.21†	1.06†	0.32
Nerve conduction velocity (m/s)	652	-0.15†	--	-0.21†	0.48†	-0.37	0.24
Short F-wave latency (ms)**	544	-0.15†	--	-0.40†	0.95†	0.12	0.36
Quantitative functional PNS tests							
Hand strength summary z-score	671	-0.03†	0.03†	0.02†	--	-0.54†	0.40
Sway speed - eyes open (mm/s)**	655	-0.24†	--	-0.19†	--	2.47†	0.33
Sway speed - eyes closed (mm/s)**	656	-0.40†	--	-0.42†	--	6.38†	0.31
Vibrotactile threshold summary (log µ)**	667	-0.02†	--	-0.02†	--	--	0.37

* Model R-square not reported for logistic regression models.
** Scores were multiplied by -1 so that lower scores indicate poorer test results.
† $p<0.05$

Supplemental Material, Table 4. Results from dose-response models of neurological physical examination tests among 678 male pesticide applicators

Lifetime days of use	Ankle reflex (abnormal=109, normal = 554)			Postural tremor (abnormal = 117, normal = 547)			Romberg (abnormal = 59, normal = 586)			Tandem gait (abnormal = 180, normal = 586)			Toe proprioception (abnormal = 62, normal = 603)			Toe vibration (abnormal = 120, normal = 554)		
	OR	95% CI	p-trend*	OR	95% CI	p-trend*	OR	95% CI	p-trend*	OR	95% CI	p-trend*	OR	95% CI	p-trend*	OR	95% CI	p-trend*
<u>OPs</u>																		
Acephate																		
0	1.00			1.00			--	--		1.00			--	--		1.00		
≤ median	0.51	(0.24, 1.06)	0.38	1.70	(0.98, 2.96)	0.34	--	--	--	0.58	(0.31, 1.07)	0.06	--	--	--	0.45	(0.18, 1.16)	0.27
> median	0.96	(0.45, 2.02)		1.09	(0.56, 2.12)		--	--		0.63	(0.32, 1.24)		--	--		0.72	(0.25, 2.10)	
Chlorpyrifos																		
0	1.00			1.00			1.00			1.00			1.00			1.00		
≤ median	0.93	(0.56, 1.56)	0.24	0.99	(0.61, 1.62)	0.71	1.33	(0.66, 2.65)	0.16	1.21	(0.77, 1.92)	0.39	2.20	(1.13, 4.31)	<0.01	1.52	(0.90, 2.57)	0.23
> median	0.70	(0.40, 1.24)		1.11	(0.67, 1.83)		1.68	(0.80, 3.54)		1.22	(0.74, 2.01)		2.56	(1.27, 5.13)		1.36	(0.77, 2.39)	
Coumaphos																		
0 days	1.00			1.00			--	--		1.00			1.00			1.00		
≤ median	1.42	(0.58, 3.50)	0.32	0.70	(0.27, 1.84)	0.93	--	--	--	1.08	(0.49, 2.40)	0.88	2.16	(0.90, 5.20)	0.06	1.62	(0.73, 3.60)	0.65
> median	1.40	(0.62, 3.18)		1.17	(0.54, 2.53)		--	--		1.04	(0.49, 2.21)		1.91	(0.80, 4.57)		1.01	(0.45, 2.28)	
Diazinon																		
0 days	1.00			1.00			1.00			1.00			1.00			1.00		
≤ median	1.12	(0.62, 2.00)	0.22	0.42	(0.22, 0.80)	0.21	0.85	(0.40, 1.81)	0.20	1.03	(0.61, 1.73)	0.67	0.52	(0.24, 1.12)	0.36	0.69	(0.37, 1.27)	0.36
> median	1.38	(0.83, 2.30)		0.82	(0.50, 1.33)		0.61	(0.29, 1.30)		1.11	(0.70, 1.75)		0.82	(0.43, 1.58)		1.44	(0.83, 2.50)	
Dichlorvos																		
0 days	1.00			1.00			1.00			1.00			1.00			1.00		
≤ median	0.90	(0.40, 2.03)	0.11	0.71	(0.32, 1.55)	0.61	1.36	(0.54, 3.44)	0.87	2.50	(1.32, 4.71)	<0.01	3.65	(1.80, 7.40)	<0.01	1.88	(0.95, 3.70)	0.02
> median	1.93	(0.97, 3.85)		0.93	(0.45, 1.91)		0.81	(0.30, 2.24)		2.10	(1.10, 4.00)		1.93	(0.85, 4.37)		2.00	(1.00, 4.01)	
Dimethoate																		
0 days	--**	--		1.00			--	--		--	--		--	--		--	--	
≤ median	--	--	--	1.85	(0.76, 4.52)	0.05	--	--	--	--	--	--	--	--	--	--	--	--
> median	--	--		1.96	(0.87, 4.45)		--	--		--	--		--	--		--	--	
Disulfoton																		
0 days	1.00			1.00			--	--		1.00			--	--		--	--	
≤ median	0.62	(0.23, 1.71)	1.00	2.33	(1.11, 4.90)	0.03	--	--	--	0.68	(0.30, 1.55)	0.50	--	--	--	--	--	--
> median	1.11	(0.56, 2.22)		1.68	(0.92, 3.07)		--	--		0.87	(0.46, 1.64)		--	--		--	--	

Supplemental Table 4 Continued.

Lifetime days of use	Ankle reflex (abnormal=109, normal = 554)			Postural tremor (abnormal = 117, normal = 547)			Romberg (abnormal = 59, normal = 586)			Tandem gait (abnormal = 180, normal = 586)			Toe proprioception (abnormal = 62, normal = 603)			Toe vibration (abnormal = 120, normal = 554)		
	OR	95% CI	p-trend[a]	OR	95% CI	p-trend[a]	OR	95% CI	p-trend[a]	OR	95% CI	p-trend[a]	OR	95% CI	p-trend[a]	OR	95% CI	p-trend[a]
OPs																		
Ethoprop																		
0 days	1.00			1.00			--	--		1.00			--	--		1.00		
≤ median	1.36	(0.63, 2.97)	0.85	1.96	(0.96, 4.02)	<0.01	--	--	--	1.26	(0.60, 2.65)	0.74	--	--	--	0.81	(0.28, 2.38)	0.65
> median	0.83	(0.39, 1.76)		2.33	(1.32, 4.13)		--	--		0.83	(0.43, 1.60)		--	--		1.30	(0.55, 3.07)	
Fonofos																		
0 days	1.00			1.00			1.00						1.00			1.00		
≤ median	1.16	(0.60, 2.23)	0.05	1.08	(0.60, 1.93)	0.76	0.95	(0.41, 2.23)	0.73	1.24	(0.71, 2.14)	0.52	2.77	(1.41, 5.44)	<0.01	1.91	(1.05, 3.48)	0.21
> median	1.81	(1.01, 3.21)		1.08	(0.61, 1.91)		0.86	(0.36, 2.05)		1.13	(0.65, 1.97)		3.37	(1.76, 6.48)		1.34	(0.71, 2.53)	
Malathion																		
0 days	1.00			1.00			1.00			1.00			1.00			1.00		
≤ median	1.17	(0.63, 2.18)	0.14	0.74	(0.43, 1.28)	0.80	0.73	(0.34, 1.56)	0.41	0.85	(0.51, 1.43)	0.38	1.08	(0.53, 2.19)	0.99	1.09	(0.59, 2.01)	0.33
> median	1.53	(0.84, 2.78)		1.00	(0.60, 1.69)		0.71	(0.33, 1.50)		0.79	(0.47, 1.32)		1.02	(0.50, 2.09)		1.32	(0.72, 2.41)	
Parathion																		
0 days	1.00			1.00			--	--		1.00			--	--		1.00		
≤ median	0.82	(0.39, 1.72)	0.89	0.59	(0.27, 1.28)	0.66	--	--	--	0.56	(0.28, 1.11)	0.36	--	--	--	1.21	(0.57, 2.61)	0.34
> median	1.01	(0.53, 1.95)		1.32	(0.74, 2.38)		--	--		0.87	(0.49, 1.55)		--	--		1.37	(0.69, 2.73)	
Phorate																		
0 days	1.00			1.00			1.00			1.00			1.00			1.00		
≤ median	0.68	(0.30, 1.53)	0.16	0.76	(0.37, 1.56)	0.43	1.27	(0.50, 3.23)	0.45	1.03	(0.53, 2.00)	0.78	0.95	(0.36, 2.53)	0.02	0.76	(0.36, 1.61)	0.77
> median	0.70	(0.40, 1.21)		0.84	(0.51, 1.40)		0.73	(0.35, 1.52)		1.07	(0.67, 1.71)		2.01	(1.13, 3.58)		1.11	(0.66, 1.87)	
Phosmet																		
0 days	1.00			--	--		--	--		1.00			1.00			1.00		
≤ median	2.81	(1.20, 6.63)	<0.01	--	--	--	--	--	--	1.20	(0.50, 2.86)	0.04	1.66	(0.61, 4.53)	<0.01	0.58	(0.23, 1.46)	0.53
> median	2.94	(1.29, 6.70)		--	--		--	--		2.30	(1.07, 4.96)		4.14	(1.91, 8.99)		0.90	(0.39, 2.09)	
Tebupirimfos																		
0 days	--	--		1.00			--	--		1.00			--	--		1.00		
≤ median	--	--	--	1.69	(0.70, 4.07)	<0.01	--	--	--	0.59	(0.21, 1.69)	0.65	--	--	--	0.81	(0.31, 2.14)	0.74
> median	--	--		2.75	(1.23, 6.13)		--	--		1.53	(0.64, 3.65)		--	--		0.91	(0.35, 2.38)	

Supplemental Table 4 Continued.

Lifetime days of use	Ankle reflex (abnormal=109, normal = 554)			Postural tremor (abnormal = 117, normal = 547)			Romberg (abnormal = 59, normal = 586)			Tandem gait (abnormal = 180, normal = 586)			Toe proprioception (abnormal = 62, normal = 603)			Toe vibration (abnormal = 120, normal = 554)		
	OR	95% CI	p-trend[a]	OR	95% CI	p-trend[a]	OR	95% CI	p-trend[a]	OR	95% CI	p-trend[a]	OR	95% CI	p-trend[a]	OR	95% CI	p-trend[a]
<u>OPs</u>																		
Terbufos																		
0 days	1.00			1.00			1.00			1.00			1.00			1.00		
≤ median	0.69	(0.40, 1.21)	0.44	0.91	(0.57, 1.47)	0.62	0.99	(0.50, 1.97)		0.98	(0.62, 1.56)	0.49	0.89	(0.45, 1.74)	0.11	0.84	(0.49, 1.47)	0.48
> median	1.38	(0.81, 2.35)		0.88	(0.52, 1.51)		0.56	(0.24, 1.33)		1.22	(0.74, 2.01)		1.75	(0.94, 3.24)		1.27	(0.72, 2.24)	
Tetrachlorvinphos																		
0 days	--	--		1.00			--	--		--	--		--	--		--	--	
≤ median	--	--	--	1.91	(0.67, 5.44)	0.51	--	--	--	--	--	--	--	--	--	--	--	--
> median	--	--		1.15	(0.51, 2.58)		--	--		--	--		--	--		--	--	
<u>Carbamates</u>																		
Aldicarb																		
0 days	1.00			1.00			--	--		1.00						1.00		
≤ median	0.31	(0.12, 0.84)	0.03	1.27	(0.65, 2.47)	0.27	--	--	--	1.12	(0.60, 2.10)	0.79	--	--		1.00	(0.41, 2.40)	0.71
> median	0.52	(0.19, 1.39)		1.40	(0.70, 2.77)		--	--		0.83	(0.39, 1.75)		--	--	--	1.27	(0.45, 3.56)	
Benomyl													--	--				
0 days	1.00			1.00			--	--		1.00						1.00		
≤ median	0.62	(0.26, 1.48)	0.24	1.56	(0.79, 3.06)	0.60	--	--	--	0.28	(0.12, 0.68)	0.13	--	--		0.77	(0.27, 2.17)	0.22
> median	0.69	(0.29, 1.63)		1.02	(0.49, 2.12)		--	--		0.85	(0.42, 1.72)		--	--	--	1.99	(0.83, 4.74)	
Carbaryl																		
0 days	1.00			1.00			1.00			1.00			1.00			1.00		
≤ median	0.56	(0.32, 0.98)	0.32	1.23	(0.73, 2.06)	0.10	0.64	(0.30, 1.36)	0.77	0.92	(0.57, 1.49)	0.07	0.65	(0.36, 1.18)	<0.01	1.04	(0.60, 1.82)	0.10
> median	0.77	(0.46, 1.29)		1.52	(0.92, 2.51)		0.91	(0.40, 2.06)		0.64	(0.39, 1.04)		0.27	(0.13, 0.57)		1.82	(0.94, 3.55)	
Carbofuran																		
0 days	1.00			1.00			1.00			1.00			1.00			1.00		
≤ median	0.79	(0.44, 1.43)	0.32	0.65	(0.36, 1.17)	0.56	0.40	(0.17, 0.94)	0.08	0.71	(0.41, 1.21)	0.66	1.17	(0.57, 2.39)	0.10	0.96	(0.54, 1.72)	0.16
> median	0.78	(0.45, 1.37)		1.24	(0.77, 1.99)		0.59	(0.28, 1.22)		1.19	(0.75, 1.90)		1.69	(0.92, 3.10)		1.51	(0.89, 2.56)	

OR, odds ratio; 95% CI, 95% confidence interval. Ankle reflex models were adjusted for age (years) and BMI (kg/m^2); postural tremor models were adjusted for age (years); Romberg models were adjusted for age (years), height (cm) and state; Tandem gait models were adjusted for age (years) and height (cm), Toe proprioception models were adjusted for age (years) and height (cm); Toe vibration models were adjusted for age (years), height (cm) and state.

[*] Based on the chi-square test for trend.

[**] Results from models with < 5 exposed cases are not presented.

Supplemental Table 5. Adjusted regression coefficients for quantitative functional PNS tests and pesticide use (ever-use and log_{10} lifetime days of use) among 678 male licensed pesticide applicators in the Agricultural Health Study

Exposure	Hand strength z-score (n=671)		Sway speed, eyes open (mm/s)* (n=655)		Sway speed, eyes closed (mm/s)* (n=656)		Vibrotactile threshold, (log μ)* (n=667)	
	β	95% CI	β	95% CI	β	95% CI	β	95% CI
Organophosphates								
Acephate								
Ever-use	0.10	(-0.05, 0.24)	0.53	(-0.40, 1.46)	1.05	(-0.82, 2.93)	0.01	(-0.06, 0.08)
Lifetime days	0.05	(-0.03, 0.13)	0.31	(-0.19, 0.82)	0.48	(-0.54, 1.50)	0.00	(-0.04, 0.04)
Chlorpyrifos								
Ever-use	0.10	(-0.05, 0.24)	0.53	(-0.40, 1.46)	1.05	(-0.82, 2.93)	0.01	(-0.06, 0.08)
Lifetime days	0.03	(-0.03, 0.09)	-0.23	(-0.60, 0.15)	-0.33	(-1.09, 0.42)	0.01	(-0.02, 0.05)
Coumaphos								
Ever-use	0.00	(-0.15, 0.15)	0.47	(-0.48, 1.42)	0.10	(-1.82, 2.02)	0.00	(-0.09, 0.09)
Lifetime days	-0.01	(-0.12, 0.10)	0.23	(-0.44, 0.90)	0.36	(-0.99, 1.70)	0.00	(-0.07, 0.06)
Diazinon								
Ever-use	0.03	(-0.07, 0.14)	-0.03	(-0.70, 0.64)	0.14	(-1.21, 1.49)	-0.01	(-0.07, 0.05)
Lifetime days	0.03	(-0.04, 0.10)	0.14	(-0.29, 0.58)	0.37	(-0.50, 1.25)	0.00	(-0.03, 0.04)
Dichlorvos								
Ever-use	-0.02	(-0.16, 0.12)	0.19	(-0.70, 1.08)	-0.84	(-2.64, 0.96)	-0.03	(-0.11, 0.05)
Lifetime days	-0.03	(-0.09, 0.04)	0.19	(-0.23, 0.61)	-0.17	(-1.02, 0.67)	-0.01	(-0.05, 0.02)
Dimethoate								
Ever-use	0.06	(-0.11, 0.24)	-0.27	(-1.37, 0.83)	-0.49	(-2.71, 1.72)	0.04	(-0.07, 0.14)
Lifetime days	0.02	(-0.10, 0.13)	-0.29	(-1.03, 0.45)	-0.71	(-2.20, 0.78)	0.03	(-0.03, 0.10)
Disulfoton								
Ever-use	0.08	(-0.07, 0.23)	-0.08	(-1.05, 0.90)	-1.06	(-3.02, 0.90)	0.06	(-0.02, 0.14)
Lifetime days	0.04	(-0.05, 0.14)	-0.05	(-0.68, 0.57)	-0.88	(-2.14, 0.37)	0.02	(-0.03, 0.07)
Ethoprop								
Ever-use	-0.03	(-0.17, 0.12)	-0.50	(-1.44, 0.44)	-0.83	(-2.72, 1.07)	-0.02	(-0.10, 0.06)
Lifetime days	-0.01	(-0.11, 0.08)	-0.35	(-0.96, 0.25)	-0.60	(-1.81, 0.61)	-0.01	(-0.06, 0.04)
Fonofos								
Ever-use	0.03	(-0.09, 0.16)	-0.29	(-1.08, 0.50)	-0.09	(-1.69, 1.51)	-0.02	(-0.09, 0.04)
Lifetime days	0.01	(-0.07, 0.08)	-0.14	(-0.62, 0.33)	0.01	(-0.95, 0.97)	0.01	(-0.03, 0.04)
Malathion								
Ever-use	0.10	(-0.02, 0.22)	-0.08	(-0.86, 0.69)	0.53	(-1.03, 2.09)	-0.02	(-0.09, 0.05)
Lifetime days	0.03	(-0.03, 0.10)	-0.24	(-0.63, 0.14)	-0.16	(-0.94, 0.61)	-0.02	(-0.05, 0.02)
Parathion								
Ever-use	0.04	(-0.08, 0.17)	1.09	(0.29, 1.89)	1.57	(-0.05, 3.19)	0.01	(-0.07, 0.08)
Lifetime days	0.00	(-0.08, 0.08)	0.71	(0.21, 1.21)	0.92	(-0.09, 1.93)	0.01	(-0.04, 0.06)
Phorate								
Ever-use	-0.05	(-0.16, 0.06)	0.31	(-0.41, 1.04)	0.72	(-0.74, 2.19)	0.04	(-0.02, 0.10)
Lifetime days	-0.02	(-0.09, 0.04)	0.13	(-0.30, 0.56)	0.43	(-0.45, 1.30)	0.03	(-0.01, 0.06)
Phosmet								
Ever-use	0.23	(0.08, 0.39)	-0.34	(-1.33, 0.64)	0.52	(-1.45, 2.49)	-0.06	(-0.15, 0.03)
Lifetime days	0.14	(0.05, 0.24)	-0.34	(-0.93, 0.26)	0.06	(-1.14, 1.26)	-0.04	(-0.10, 0.01)
Tebupirimfos								
Ever-use	-0.09	(-0.28, 0.09)	1.40	(0.24, 2.56)	1.19	(-1.16, 3.53)	0.04	(-0.07, 0.14)
Lifetime days	-0.05	(-0.16, 0.06)	0.82	(0.10, 1.53)	0.73	(-0.72, 2.18)	0.03	(-0.03, 0.09)
Terbufos								
Ever-use	0.06	(-0.04, 0.17)	0.05	(-0.73, 0.62)	-1.07	(-2.43, 0.29)	0.02	(-0.04, 0.08)
Lifetime days	0.04	(-0.02, 0.09)	-0.10	(-0.46, 0.26)	-0.57	(-1.29, 0.15)	0.00	(-0.03, 0.03)

Table 5. Continued.

Exposure	Hand strength z-score (n=671)		Sway speed, eyes open (mm/s)* (n=655)		Sway speed, eyes closed (mm/s)* (n=657)		Vibrotactile threshold, (log µ)* (n=667)	
	β	95% CI	β	95% CI	β	95% CI	β	95% CI
Tetrachlorvinphos								
Ever-use	0.10	(-0.08, 0.27)	-0.19	(-1.32, 0.94)	-0.98	(-3.26, 1.31)	-0.01	(-0.11, 0.09)
Lifetime days	0.05	(-0.06, 0.15)	-0.09	(-0.78, 0.61)	-0.39	(-1.79, 1.01)	0.00	(-0.06, 0.06)
<u>Carbamates</u>								
Aldicarb								
Ever-use	-0.01	(-0.16, 0.13)	0.99	(0.07, 1.91)	2.47	(0.62, 4.32)	0.11	(0.04, 0.19)
Lifetime days	-0.01	(-0.08, 0.09)	0.62	(0.09, 1.16)	1.17	(0.10, 2.24)	0.05	(0.01, 0.10)
Benomyl								
Ever-use	0.04	(-0.10, 0.19)	0.61	(-0.33, 1.54)	1.39	(-0.50, 3.28)	0.04	(-0.04, 0.12)
Lifetime days	0.00	(-0.10, 0.09)	0.33	(-0.28, 0.95)	0.32	(-0.91, 1.55)	0.02	(-0.03, 0.08)
Carbaryl								
Ever-use	0.04	(-0.08, 0.16)	-0.43	(-1.18, 0.32)	0.23	(-1.30, 1.76)	-0.04	(-0.10, 0.03)
Lifetime days	0.04	(-0.02, 0.11)	-0.13	(-0.55, 0.30)	0.34	(-0.52, 1.21)	-0.01	(-0.04, 0.03)
Carbofuran								
Ever-use	0.04	(-0.06, 0.15)	0.27	(-0.39, 0.93)	0.17	(-1.17, 1.50)	0.02	(-0.04, 0.08)
Lifetime days	0.04	(-0.03, 0.10)	0.04	(-0.38, 0.46)	-0.27	(-1.11, 0.57)	0.02	(-0.02, 0.06)
<u>Summary variables</u>								
Lifetime days to ALL OPs	0.01	(-0.06, 0.07)	0.13	(-0.34, 0.61)	0.20	(-0.76, 1.16)	0.02	(-0.03, 0.06)
Lifetime days to ALL pesticides	0.06	(-0.04, 0.17)	0.22	(-0.45, 0.89)	0.29	(-1.06, 1.64)	0.03	(-0.03, 0.09)
HPEEs (ever)	0.14	(0.02, 0.26)	0.37	(-0.40, 1.14)	0.96	(-0.60, 2.51)	0.01	(-0.06, 0.08)

HPEEs, high pesticide exposure events. Hand strength models were adjusted for age (years), BMI (kg/m^2), height (cm) and state; sway speed models (with both eyes open and closed) were adjusted for age (years), height (cm) and state; vibrotactile threshold models were adjusted for age (years) and height (cm).

* Scores have been multiplied by -1 so that lower scores indicate poorer test performance.
